# Ion Channels Involved in Tooth Pain

**DOI:** 10.3390/ijms20092266

**Published:** 2019-05-08

**Authors:** Kihwan Lee, Byeong-Min Lee, Chul-Kyu Park, Yong Ho Kim, Gehoon Chung

**Affiliations:** 1 Gachon Pain Center and Department of Physiology, College of Medicine, Gachon University, Incheon 406-799, Korea; key1479@gmail.com (K.L.); pck0708@gachon.ac.kr (C.-K.P.); 2 Department of Oral Physiology and Program in Neurobiology, School of Dentistry, Seoul National University, Seoul 08826, Korea; bmjj88@snu.ac.kr; 3 Dental Research Institute, Seoul National University, Seoul 03080, Korea

**Keywords:** tooth pain, TRP channels, odontoblasts, piezo, purinergic, trigeminal ganglion

## Abstract

The tooth has an unusual sensory system that converts external stimuli predominantly into pain, yet its sensory afferents in teeth demonstrate cytochemical properties of non-nociceptive neurons. This review summarizes the recent knowledge underlying this paradoxical nociception, with a focus on the ion channels involved in tooth pain. The expression of temperature-sensitive ion channels has been extensively investigated because thermal stimulation often evokes tooth pain. However, temperature-sensitive ion channels cannot explain the sudden intense tooth pain evoked by innocuous temperatures or light air puffs, leading to the hydrodynamic theory emphasizing the microfluidic movement within the dentinal tubules for detection by mechanosensitive ion channels. Several mechanosensitive ion channels expressed in dental sensory systems have been suggested as key players in the hydrodynamic theory, and TRPM7, which is abundant in the odontoblasts, and recently discovered PIEZO receptors are promising candidates. Several ligand-gated ion channels and voltage-gated ion channels expressed in dental primary afferent neurons have been discussed in relation to their potential contribution to tooth pain. In addition, in recent years, there has been growing interest in the potential sensory role of odontoblasts; thus, the expression of ion channels in odontoblasts and their potential relation to tooth pain is also reviewed.

## 1. Introduction

The tooth is a unique sensory system that senses external stimuli predominantly as nociception. Most of the nerves innervating tooth pulp have been presumed to be nociceptors since most axons in tooth pulp are unmyelinated or small fibers that are myelinated [1]. However, this belief was challenged by multiple observations that pulpal nerves possess physical and chemical properties of large myelinated Aβ fibers. Due to these paradoxical findings, a new concept of “algoneurons” was introduced [2,3].

**Figure 1:**
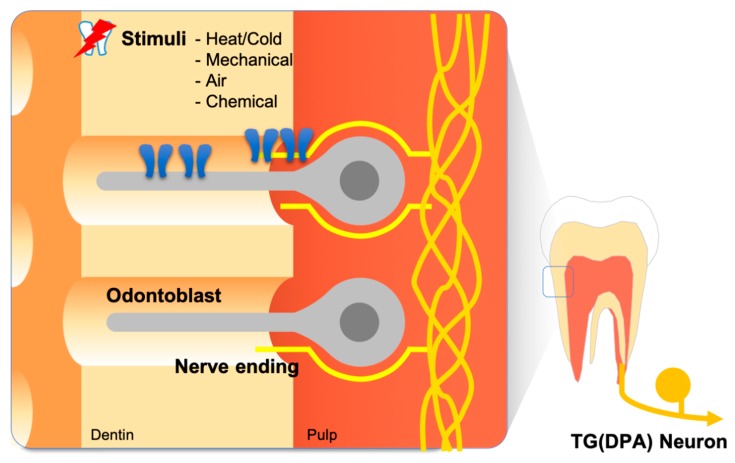
Anatomical features of the dental pain sensory system. Odontoblasts comprise the outermost cell layer of dental pulp tissue, which is advantageous to odontoblasts playing the role of a sensory transducer. Some nerve endings of dental primary afferents (DPAs) spread into the dentinal tubule. This structural nature establishes a distinctive sensory mechanism for the tooth.

The structure of the tooth is comprised of densely vascularized and innervated tooth pulp covered by two layers of hard tissue—the dentin and enamel [3,4]. The dentin and enamel are distinguished by their microstructure and mineral content. The outermost enamel layer is the hardest tissue in the body, with minerals forming 97% of its weight. The dentin layer lies between the tooth pulp and the enamel layer and has an intermediate hardness with a mineral content slightly higher than that of bone, providing resilience to the enamel. The most notable property of dentin is its microstructure. Dentin is made of thousands of microtubules—dentinal tubules—filled with dentin tubular fluid. Odontoblasts are the cells that deposit the calcium matrix to form dentin and constitute a cellular single layer at the inter-surface of the dentin and the tooth pulp. Each odontoblast possesses a process that protrudes into the dentinal tubules (Figure 1).

The structure of teeth results in a unique pattern of nociception. One example is a special condition known as dentin hypersensitivity—the exaggerated nociception in teeth caused by non-noxious mechanical, chemical, or thermal stimuli without the pulpal inflammation predisposed or the nerve damage in the adjacent tissue [5,6,7,8]. While the molecular mechanisms underlying dentin hypersensitivity have not been fully elucidated, one promising hypothesis—the hydrodynamic theory—states that external stimuli cause the movement of the dentin tubular fluid to, ultimately, excite nerve fibers in the pulp to initiate pain. This provides the most plausible explanation for dental cold hypersensitivity of all the hypotheses that have been proposed, although not without controversy [9,10,11,12,13,14,15,16,17,18,19,20]. Another example is the pulsating nature of tooth pain often described by chronic pulpitis patients. This phenomenon is presumed to be caused by hydrostatic pressure applied to the edematous tooth pulp in the restricted space within the dentin and enamel. Both the pulsating pain associated with pulpal inflammation and the hydrodynamic theory of dental hypersensitivity require a mechanosensitive receptor as a key molecule. However, understanding such a receptor and its associated mechanism of action only began not long ago. This review summarizes the most recent advances in the understanding of the molecular and cellular mechanisms of mechanotransduction in the context of tooth pain.

The tooth is exposed to drastic temperature changes of the oral cavity. Although the harsh thermal conditions from food consumption hardly induce tooth pain under normal circumstances because of the excellent thermal insulating of the enamel tissue [21,22,23,24], mild temperature changes can induce intense pain with exposed dentin or pulpal inflammation. For example, noxious cold induces sharp and transient pain while noxious heat induces dull and lasting pain [25,26]. To elucidate the molecular mechanisms associated with temperature-driven tooth pain, the expression and physiology of molecular thermosensor candidates, such as the transient receptor potential (TRP) channel superfamily, have been investigated. A large variety of temperature receptors that may play critical roles in the transduction of tooth pain are expressed in dental primary afferent nerves [22,27] and odontoblasts [5,6,8,9,10,18].

In addition, voltage-gated and ligand-gated ion channels take important roles in tooth pain. Not only are various types of voltage-gated ion channels expressed in the trigeminal sensory nerve on common nerve cells, but they are also expressed in odontoblast cells [22,28,29,30,31,32]. Previous studies have indicated that small molecules, such as adenosine 5′-triphosphate (ATP), and their ionotropic receptors, the P2X family, play an important role in the sensory system for tooth pain [19,33,34,35]. In this review, we summarize the research on temperature-sensitive, mechanosensitive, ligand-gated, and voltage-gated ion channels and their role in the sensory system for tooth pain.

## 2. Thermo-Sensitive Ion Channels

**Figure 2:**
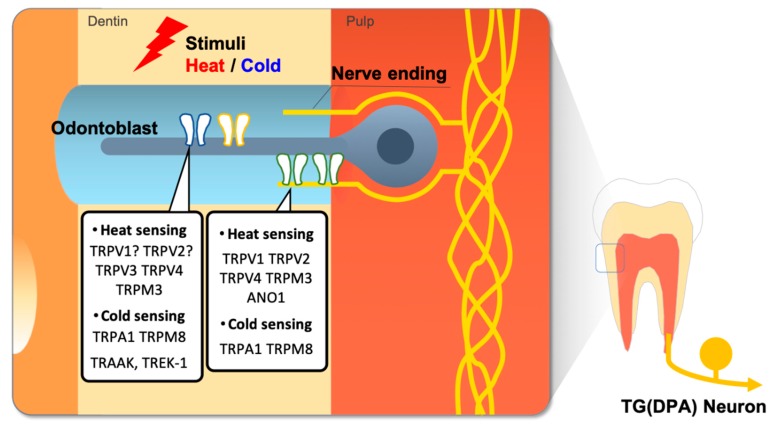
Thermosensitive ion channels in the dental sensory system. External heat or cold stimuli cause activation of thermosensitive ion channels in dental primary afferent (DPA) nerve ending or odontoblast cells, therefore dental pain transduces from thermal stimuli.

Since the temperature-gated TRPV1 ion channel was first cloned from a subset of trigeminal and dorsal root ganglia (DRG) neurons [36,37], several members of the TRP superfamily have been discovered and proposed as potential molecular temperature sensors [38]. These TRP channels have been hypothesized to be key contributors for the keen sense of temperature in teeth, and the functional expression of TRP channels in dental primary afferent neurons and in odontoblasts has been massively investigated (Figure 2) [24,26].

### 2.1. Thermo-Sensing Ion Channels in the Trigeminal Nerve

TRPV1 is a polymodal receptor activated by high temperatures over 43 °C or irritant chemicals including capsaicin and proton. TRPV1 is believed to play a central role in nociception because it is primarily expressed in small- to medium-peptidergic nociceptive neurons, and its activation is modulated by various inflammatory and nerve-damage-inducing mediators. Immunohistochemical investigation demonstrated TRPV1 expression in 20% of rat trigeminal ganglion (TG) cells, mostly in small- to medium-sized, as expected [39]. Interestingly, a retrograde labeling study revealed that only 8% of tooth pulpal neurons were TRPV1-positive, whereas 26% of TG neurons innervating facial skin were TRPV1-positive, which was contrary to the previous speculation that most nerves innervating the tooth pulp are nociceptive [40,41,42]. Conversely, the functional analysis of retrograde-labeled dental primary afferent neurons showed the opposite results; the neuron response to capsaicin application was more abundant for dental primary afferents than for TG neurons in calcium imaging studies [43] and in whole-cell patch clamp experiments [44]. Single-cell RT-PCR analysis revealed that most dental primary afferents are TRPV1-positive, whereas two other immunohistochemical analyses reported that only 17–34% of pulp-innervating neurons were TRPV1-positive [45,46]. The reason for this discrepancy is not clear. Of note, lipopolysaccharides (LPS) from Gram (−) bacteria upregulated TRPV1 expression in TG [47], and Complete Freund’s Adjuvant (CFA) upregulated TRPV1 in TG neurons innervating adjacent teeth [48], suggesting the potential contribution of TRPV1 to tooth pain under the pulpitis condition. Interestingly, estrogen is also upregulated TRPV1 and anoctamin-1 (ANO1)—a potential heat-sensing ion channel—in female rat TG neurons and induced an increased pain response to TRPV1 agonists [49]. The physiological meaning of estrogen-induced upregulation for heat sensing ion channels is not clear and needs to be considered when designing pain studies.

TRPV2 is an ion channel homolog to TRPV1 with a higher threshold (>52 °C). TRPV2 is different from TRPV1 in that it does not respond to capsaicin nor acid and is preferentially expressed in medium- to large-sized myelinated neurons [50]. The immunohistochemical analysis of retrograde-labeled TG neurons revealed a TRPV2 expression pattern quite the opposite to that of TRPV1 [51]. While 14% of the TG cells showed immunoreactivity to TRPV2 mostly in medium- to large-sized, 37% of neurons innervating tooth pulp was TRPV2-positive, whereas only 9% of neurons to the facial skin were positive. Another immunohistochemical analysis using a double-labeling technique confirmed the mutually exclusive expression of TRPV1 and TRPV2 in pulpal neurons, with 32–51% TRPV2 positive cells [45,46]. These findings are consistent with previous reports that pulpal neurons are mostly medium- to large-sized myelinated neurons that lose their myelination upon entering tooth pulp [2,4,52,53,54,55,56], suggesting that teeth might have a distinct nociceptive system.

In addition, the expression of TRPV4 and TRPM3 was observed in retrogradely labeled dental afferent neurons [57,58,59]. Because TRPV4 activates at innocuously warm temperature between 27 and 35 °C, it is believed to play a role in the maintenance of body temperature, rather than in nociception [60]. On the other hand, TRPM3, or long TRPC3 as previously known, was recently discovered to have an activation threshold of 40 °C and became a prominent candidate of noxious heat detector [61]. 

Since cold stimuli induce tooth pain more frequently than hot, cold-sensitive TRP channels might play a role in the transduction of tooth pain. TRPA1 and TRPM8 are cold-sensitive TRP channel subtypes activated at temperatures below 17 and 25 °C, respectively [62,63]. Calcium imaging experiments with cold stimuli under 18 °C revealed that cold-sensitive neurons are more abundant in the TG than in the DRG (15% and 7%, respectively) [43]. TRPA1 upregulation in a tooth injury rat model proposes the importance of TRPA1 in tooth pain [64]. A subsequent study combining electrophysiological recording with single-cell RT-PCR and immunocytochemistry revealed the functional expression of TRPA1 and TRPM8 in rodent dental primary afferent neurons [43]. Interestingly, the expression of TRPA1 and TRPM8 channels was lower than that of TRPV1 in dental primary afferent (DPA) neurons. TRPA1 and TRPM8 were, moreover, co-expressed in some of the TRPV1-positive DPA neurons, suggesting an ambiguity between cold and hot stimuli-induced tooth pain. A recent study suggested that acute heat sensation requires any of functional TRPV1, TRPA1, and TRPM3 ion channels, and only triple knock-out mice showed a lack of acute withdrawal response to noxious heat compared to the intact normal response to cold stimuli, which suggests a redundant mechanism for heat detection [65]. Whether dental sensory systems utilize a similar mechanism is unclear.

### 2.2. Thermo-Sensing Ion Channels in Odontoblast Cells

Odontoblasts deposit calcium matrix at the outer surface of tooth pulp to form the dentin layer. Due to this anatomical location, the potential secondary role of odontoblasts as a member of the sensory system has been continuously proposed [3,21,22,66,67,68,69,70,71,72,73,74]. The expression of temperature-sensing TRP channels in odontoblasts has been investigated by several researchers, but the results have been diverse. While calcium imaging, immunohistochemical detection, and single cell RT-PCR all revealed the negative expression of heat-sensing TRPV1 and TRPV2 channels in acutely isolated odontoblasts from adult rat incisors [71], calcium imaging and electrophysiological recording of the odontoblasts cultured from neonatal rat pulpal slices showed positive responses to TRPV1, TRPV2, TRPV3, TRPV4, and TRPM3 [69,72]. It was not clear whether TRPV1 or TRPV2 channel-expressing odontoblasts were damaged or lost during acute isolation, whether the odontoblasts cultured from pulpal slices did not faithfully reflect the naïve odontoblasts, or whether it was from the age difference. Results from cold-sensing TRPA1 and TRPM8 investigation are more perplexing. While TRPA1 and TRPM8 were not detected in both acutely isolated odontoblasts and in pulpal slice-derived odontoblasts [71,72], another study showed both TRPA1 and TRPM8 in rat odontoblasts cultured from pulpal slices [70]. 

The results from human odontoblasts are less diverse. TRPV1-4 and TRPM8 have been detected by functional, immunohistochemical, western-blotting and electron microscopic tests [66,67,75]. TRPA1, however, showed controversial results. While in one study [67] immunohistochemical analysis of decalcified healthy human molar sections detected TRPA1 expression, another study did not [68]. Further clarification is required to determine the expression of TRPA1 in human odontoblasts. Nonetheless, it is very probable that odontoblasts functionally express temperature-sensing TRP channels and that these channels might confer odontoblasts with the ability to detect hot and cold temperatures. Many questions remained to be answered, including whether odontoblasts, if activated, can transfer these signals to pulpal neurons.

### 2.3. Other Aspects of the Thermo-Sensing Ion Channels in the Dental Sensory System

TRAAK and TREK-1 channels are also considered as potent thermosensitive ion channels [32,38]. Noël and his colleagues demonstrated that TRAAK and TREK-1 participate in the heat and cold sensing functions of TRP channels [76]. Their expression in odontoblast cells was demonstrated in a rodent model and in human pulp tissue [8,77]. Many other ion channels, including voltage-gated Na^+^ (Na_V_) channels (VGSCs), have been thoroughly studied as molecular thermosensors [78,79]. Recently, other types of dental cells, such as human tooth pulp fibroblasts and periodontal ligament (PDL) cells, were also shown to express temperature sensitive TRP channels [80,81]. These findings suggest that apart from odontoblasts, other cell types, such as pulp fibroblast cells or PDL cells, might contribute to the response to noxious thermal stimuli. Further studies are needed to elucidate the thermosensing mechanisms of various cell types surrounding tooth tissue. Alternatively, some efforts to characterize the dental sensory system by Next Generation Sequencing (NGS) studies have also been performed [82,83]. Combining these results with new emerging experimental methodologies, such as NGS or multi-omics studies of dental sensory systems, understanding of the temperature-induced tooth pain perception mechanisms may prove to be a significant scientific breakthrough. 

## 3. Mechanosensitive Channels in Tooth Pain

**Figure 3:**
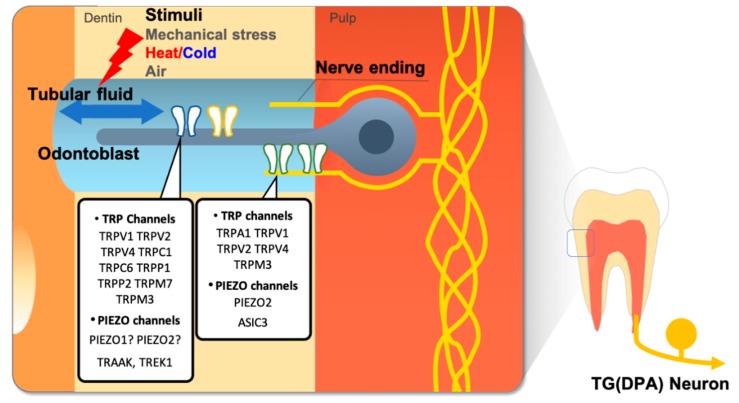
Mechanosensitive ion channels in the dental sensory system. According to the hydrodynamic theory of dental nociception, movement of the dentine tubular fluid generated by external stimuli, such as thermal or mechanical stress, activates mechanosensitive ion channels in odontoblasts or dental primary afferent (DPA) nerve ending extend into the dentinal tubule. Thus, mechanosensitive ion channels are regarded as major players in dental nociception. These ion channels can also be activated with directly applied mechanical stress.

It is difficult to explain tooth pain strictly by transduction of noxious temperatures by thermo-TRP channels. Temperature transduction cannot explain the sudden and intense tooth pain elicited by innocuous stimuli, such as an air puff, water spray, or sweet substances, or the pulsating pain often described by chronic pulpitis patients. Evidence from clinical studies suggests that the movement of dentin tubular fluid by temperature change might cause the sudden intense tooth pain from an air puff or spray of water. The sudden intense pain can also be generated in the micro-movement of cracked tooth parts during mastication. In addition, tooth structure can be mechanically deformed in response to thermal changes [3,13,14,15,16]. Pulsating pain in chronic pulpitis results from hydrostatic blood pressure applied to inflamed and swollen pulp tissue contained within the hard dentin structures [84,85]. All of these are suggested molecular transducers of mechanical force or stretch expressed in the dental sensory system, that are activated upon mechanical stimulation from movement of dentinal fluid, or deformation of microstructure (Figure 3) [13,18].

### 3.1. TRP Channels

Several TRP channel superfamily members that exhibit mechanosensitivity include TRPC1, TRPC6, TRPV1, TRPV2, TRPV4, TRPM3, TRPM4, TRPM7, TRPA1, and TRPP2 [86]. Of these channels, the expression of TRPV1, TRPV2, TRPV4, TRPM3, TRPM7, and TRPA1 was reported in TG neurons [59,87,88], while TRPV1, TRPV2, TRPV4, TRPM3, and TRPA1 were shown in dental afferent neurons with retrograde labelling [43,57,58,59,72,89,90].

TRPV1, although this is still in debate, has been proposed to have mechanosensitivity. Bladder and urothelial epithelial cells from TRPV1-deleted mice showed markedly diminished responses to stretch [91]. The expression of TRPV1 in TG neurons innervating tooth pulp or in odontoblasts is also controversial, as elaborated in the previous section. The mechanosensitivity of TRPA1 is similar. While TRPA1-deleted mice showed a higher threshold and reduced response to mechanical stimuli [92], another TRPA1-null mouse line reported no difference in mechanical threshold compared to wild-type mice [89]. Ex vivo skin-nerve recordings from TRPA1-null mice showed deficits in mechanical sensitivity [93]. Although the role of TRPA1 as a cellular mechanical transducer is unclear, it suggests that TRPA1 may be implicated in mechanical hyperalgesia under pathological conditions. A recent report on the upregulation of TRPA1 in an experimental tooth injury model suggests that TRPA1 is still a promising candidate transducer in teeth [94].

TRPV4 is expressed in many cell types and tissues where mechanosensitivity is critical, such as hair cells of the inner ear, vibrissae Merkel cells, sensory ganglia, chondrocytes, osteoclasts, osteoblasts, and keratinocytes, as well as cutaneous A- and C-fiber terminals [95]. Studies conducted in TRPV4-null mice revealed that TRPV4 is related to the development of acute inflammatory mechanical hyperalgesia [95,96,97]. TRPV4-deleted mice showed reduced C-fiber sensitization for mechanical and hypotonic stimuli [98], suggesting TRPV4 involvement in osmotic mechanical hyperalgesia and nociceptor sensitization [98,99]. Recently, one study showed TRPV4 expression in the nerves of human tooth pulps and that TRPV4 expression was upregulated in human tooth pulp nerves of symptomatic teeth associated with pulpitis [100].

The investigation of non-neuronal cells revealed the expression of TRPC1, TRPC6, TRPV4, TRPM3, TRPM7, TRPP1, and TRPP2 in rodent odontoblasts [72,90,101,102] and TRPV1, TRPV2, TRPV4, and TRPM3 in pulp cells from neonate rats after in vitro differentiation into odontoblasts [72]; this suggests that these channels might function as molecular mechanotransducers that possibly confer mechanosensitivity to odontoblasts. TRPM7 is a unique ion channel with mechanosensitivity attached to a kinase, as shown by a touch-unresponsive zebrafish mutant [103]. Interestingly, TRPM7 expression was detected in most odontoblasts, predominantly in the odontoblastic process region [101], and TRPM7-specific inhibitor blocked mechanically-evoked calcium responses in odontoblasts [101], suggesting that TRPM7 might mediate mechanical sensitivity in odontoblasts. TRPP1 and TRPP2, which act together as a mechanical receptor, are present on the surface of odontoblasts and appear to be located at the base of the primary cilium [104]. 

Recent publications strongly suggest that IB4-positive non-peptidergic afferents play an important role transducing mechanical stimuli in the skin [105,106]. Chung and his colleagues showed a non-peptidergic mechanosensitive subpopulation in TG neurons that might be responsible for the detection of dentin tubular fluid [107]. However, the mechanical transducer molecule responsible for tooth pain in non-peptidergic polymodal nociceptors remains to be elucidated by future research.

### 3.2. PIEZO Channels

Since PIEZO family ion channels were cloned in mammals, the PIEZO gene family have been considered as putative mechanosensitive ion channel proteins [108,109,110,111,112,113,114]. PIEZO1 and PIEZO2 were identified by efforts to elucidate mammalian mechanosensing mechanisms which could not be clearly understood by TRP channels. While PIEZO channels are broadly expressed in a wide range of mammalian mechanosensitive cell types, PIEZO2 channels are identified as low-threshold mechanoreceptors in sensory DRG neurons and Merkel cells [115,116,117]. Moreover, the depletion of PIEZO2 in sensory DRG neurons and Merkel cells resulted in the dramatic reduction of rapidly adapting mechanically induced currents, suggesting a critical role of PIEZO2 as low threshold mechanoreceptor [118]. These findings have great implications for tooth pain research because low threshold mechanoreceptors are regarded as major players in tooth pain sensory systems, considering that mild mechanical stimuli could cause severe tooth pain. Moreover, several studies revealed that major populations of dental primary afferent neurons consist of A-fibers regarded as low-threshold mechanoreceptors [2,53]. Recently, many groups have examined the functional expression of PIEZO2 in the dental sensory system. Won et al. demonstrated PIEZO2 expression in murine dental primary afferent neurons by single-cell RT-PCR and in situ hybridization and function by recording rapidly adapting inward current induced by direct pocking [119]. Interestingly, PIEZO2 positive dental primary afferent neurons were medium-to-large sized and co-expressed with TRPV1, Nav1.8, and CGRP, which are regarded as nociceptive neuronal marker genes. These results indicate that PIEZO2 positive low-threshold mechanoreceptor neurons innervating teeth are ‘algoneurons’ that also, paradoxically, act as nociceptors. 

To verify the role of these low-threshold mechanoreceptors in the odontoblast cells, other studies have been performed to verify PIEZO expression in odontoblast cells. An electrophysiological study with odontoblast cells co-cultured with IB4-negative medium-sized TG neurons elucidated the role of odontoblasts as mechanosensitive transducer cells [120]. Inward currents were detected from TG neurons when mechanical stimulation was applied to neighboring odontoblast cells. Interestingly, this odontoblast-induced inward current from TG neurons was antagonized with a PIEZO1 selective blocker. Three-dimensional imaging with focused ion beam-scanning electron microscopy revealed that PIEZO2 is expressed in nearly all rodent matured odontoblast cells and is absent in immature cells [121]. PIEZO2 proteins were detected selectively in odontoblastic processes that protrude into dentinal tubules. In another study, however, odontoblastic response to mechanical stimulation was inhibited by a specific antagonist of PIEZO1 [120]. These controversial results indicate the essential role of PIEZO ion channels in dental sensory systems as putative mechanosensors but also suggest that more research is needed to comprehensively understand complex dental mechanosensing systems.

### 3.3. ASIC Channels

Acid-sensing ion channels (ASICs) were initially implicated in mechanotransduction because their phylogenetic homologs in *Caenorhabditis elegans*—the mechanosensory (MEC) channel subunits—are essential for the perception of touch. Three members of the ASIC family (ASIC1-3) are expressed in peripheral mechanoreceptors and nociceptors in mammals. Six ASIC proteins encoded by four genes have been identified, ASIC1a, ASIC1b, ASIC2a, ASIC2b, ASIC3, and ASIC4, which differ in their kinetics, external pH sensitivity, tissue distribution, and pharmacological properties [122]. ASIC-2 mRNA is expressed in both small-diameter and large-diameter neurons and colocalized within single sensory neurons in the TG [123]. One-third of TG neurons that project towards the tooth pulp are immunoreactive (IR) to ASIC3 [124]. A single-cell RT-PCR study revealed that the ASIC3 mRNA is expressed in 67% of pulpal afferent neurons [58,59]. Human odontoblasts display immunoreactivity for ASIC2 as well as the ENaC-β and ENaC-γ, but not the ENaC-α, subunits [125]. These findings suggest a role for ASIC3 in the mechanotransduction of tooth sensitivity.

### 3.4. TREK-1 Potassium Channels

The primary function of the two-pore potassium (K_2P_) channels is to mediate K^+^-selective leak currents that regulate cell excitability through a hyperpolarized resting membrane potential [126]. Several members of the K_2P_ channel family including TRESK, TRAAK/KCNK4, TASK, TREK, and THIK are intrinsically mechanosensitive, and all are expressed in the DRG [127,128]. K_2P_ channels are well established regulators of primary afferent fibers excitability. Two kinds of high conductance Ca^2+^-activated K^+^ (K_Ca_) channels and TREK-1 channels (TWIK-related K^+^ channels) have been identified as putative mechanotransduction channels [19,46,102,113,114,129,130,131]. Investigation of K_2P_ ion channels in the mammalian tooth pulp and in the odontoblast membrane revealed TREK1 mRNA expression in human odontoblasts [58,77]. Consequently, TREK-1 channels when stretch-activated may participate in the signal transduction to afferent nerve endings.

## 4. Ligand-Gated Channels

### ATP: Purinergic Receptors

ATP acts as an extracellular signaling molecule that affects numerous downstream factors and signaling cascades. Signaling involving a purine nucleotide or nucleoside, such as ATP, is called purinergic signaling and is associated with multiple levels of nociception and immune responses in the oral system [132]. For example, P2X receptors (P2XRs) are expressed in the nociceptive TG cells [133,134] as well as in tooth pulp cells [35,135,136]. P2X positive nerve fibers have been detected in the subodontoblastic plexus close to odontoblasts [33,135,137]. P2XR2 and P2XR3 receptors have been found in both pulp nerves and a subpopulation of rat TG neurons [134,138,139,140,141]. In addition, a study showed that the presence of the P2X3 receptor and possibly the heteromeric P2X2/3 receptor in the trigeminal subnucleus caudalis (Vc) initiates and maintains the central sensitization in rat tooth pulp nociceptive neurons [142].

**Figure 4:**
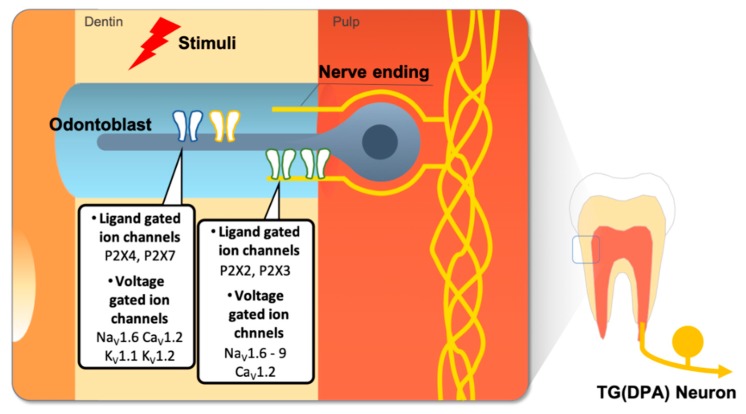
Other types of ion channels such as ligand gated ion channels and voltage gated ion channels expressed in the dental sensory system. ATP molecules released by adjacent odontoblast or fibroblast cells in pulp by external stimuli and they induce activation of purinergic receptors in odontoblasts or DPA neurons. Various types of voltage gated ion channels are also expressed in dental pain sensory cells but their functions are not clearly revealed.

Recent studies suggest that P2X3 receptor activation by ATP induces tooth nociception in rat tooth pulp [33,139]. Importantly, an ATP derivative is sufficient to elicit behavioral pain sensation in tooth pulp [143] and odontoblasts contribute to the sensory function of teeth by releasing ATP in response to physical stimuli [19,66,129,144,145]. Furthermore, odontoblasts themselves express different P2XR subtypes (Figure 4) [34,146]. Since blocking extracellular ATP release results in the inhibition of interodontoblastic communication, ATP might regulate the physiology of odontoblasts via autocrine or paracrine mechanisms [19]. G-protein coupled P2Y ATP receptors are also present in pulp cells [136], TG neurons [147,148], trigeminal satellite glial cells [149], and odontoblasts [129,136]. 

## 5. Voltage-Gated Ion Channels

### 5.1. Voltage-Gated Sodium Channels

VGSCs are responsible for action potential generation and excitability of the cell membrane. Nine different VGSC isoforms have been discovered in the mammalian nervous system, with Na_V_1.6 and Na_V_1.7 being the most abundant in the peripheral nervous system [150] and nociceptive sensory neurons [151], respectively. Immunohistochemical analysis of pulp tissue taken from pulpitis patients revealed expression of Na_V_1.7 and Na_V_1.8 with greater immunoreactivity in the pulp from patients with painful pulpitis [152,153,154]. Closer investigation of tooth pulp from pulpitis patients showed an increased expression of Na_V_1.7 in the nerve bundles at intact and demyelinating nodes of Ranvier compared with healthy tooth pulp [155], while no significant difference for Na_V_1.6 expression was observed [156]; together, this suggests that Na_V_1.7 might play a role in inflammatory tooth pain.

Since expression of VGSCs is an important property of excitable cells, the demonstration of Na_V_1.6 expression in non-neuronal pulpal cells, such as pulpal immune cells, dendritic pulpal cells, and odontoblasts [29], has gathered a robust interest. In addition, electrophysiology, immunohistochemistry, RT-PCR, and in situ hybridization of odontoblasts differentiated from human dental pulp explants has revealed the expression and functionality of Na_V_1.1, Na_V_1.2, and Na_V_1.3 [28]. Interestingly, patch-clamp recording of the cultured human tooth pulp cells revealed rapidly inactivating TTX-sensitive Na^+^ currents and membrane properties similar to neuronal satellite cells but not to odontoblasts [157]. The molecular and cellular identity of such pulpal cells is still unknown, and whether odontoblasts or other pulpal cells are indeed excitable and, if so, what their function would be, is unclear.

Na_V_1.9 is the VGSC most recently identified [158]. Na_V_1.9 is preferentially expressed in small-diameter DRG neurons, TG neurons, and myenteric neurons [159,160,161,162] Na_V_1.9 is activated at voltages near the resting membrane potential and generates a relatively persistent current [159]. Na_V_1.9 channels may also have a role in inflammatory pain, but not in neuropathic pain [158,163]. In addition, an investigation of Na_V_1.9 in rats revealed the innervation of Na_V_1.9-IR fibers in lip skin and in the tooth pulp of non-painful teeth, suggesting a role of this VGSC isoform in orofacial pain [164]. Recently, a study found that Na_V_1.9 was increased in the axons of symptomatic pulpitis of permanent painful human teeth compared to the tooth pulp of permanent non-painful teeth (Figure 4) [165].

### 5.2. Voltage-Gated Calcium Channels

Several lines of evidence have shown that DRG and spinal cord neurons express Ca_V_1.2 [166], while L-type Ca_V_ channels are broadly expressed in skeletal and cardiac muscle, neurons, auditory hair cells, pancreatic cells, and the retina [167]. Electrophysiological examination of the DRG showed that L-type Ca_V_ channels are present largely in small and large neurons, although these channels are regulated during chronic pain [168]. One study using RT-PCR showed that L-type Ca_V_ channels are downregulated in DRG upon chronic constriction injury (CCI) and sciatic nerve axotomy in rats [169]. suggesting that decreases in Ca_V_1.2 and Ca_V_1.3 in DRG could contribute to the hyperexcitability of neuropathic pain by modulating Ca^2+^-dependent inactivation or facilitation as negative feedback [170]. Inversely, Ca_V_1.2 is upregulated in the spinal cord in a spinal nerve ligation (SNL) model. One study reported that Cav1.2 functions as a key factor for the differentiation of tooth pulp stem cells [171]. In addition, several lines of evidence indicate that Ca_V_1.2 may have a central role in odontoblast behavior at both the physiological and pathological levels [31,172,173,174].

### 5.3. Voltage-Gated Potassium Channels

Patch-clamp recording revealed the presence of the voltage-gated potassium channel (K_V_ channel) in cultured human dental pulp cells [175] and in human odontoblasts [73]. Calcium-activated potassium (K_Ca_) channels that display mechanosensitivity are also present in odontoblast cells [30,31,176], and their concentration at the apical pole of odontoblasts could have relevance in the sensory transduction process of teeth [73]. 

## 6. Conclusions

Tooth pain greatly undermines patient quality of life. Tooth pain arises from distinct mechanisms from other pain types because of the unique neurochemical properties and anatomical structure of dense innervation and vascularization under hard tissue. The physiology of tooth pain involves the complex orchestration of ion channels introduced in this review (Table 1). Still, the present understanding is vague. Many questions remain, such as how mechanosensitive ion channels involved in tooth pain are molecular identified, whether odontoblasts function as primary sensory cells, and, if so, how they provide signals to underlying nerves. Elucidating these questions will provide the basis for understanding tooth pain and can lead to the development of therapeutics specifically targeting tooth pain.

## Figures and Tables

**Table 1:** Tabular summary of ion channels expressed in dental pain sensory system and their functions.

Ion Channel Type	Cell Type	Expressed Ion Channels	Remarks
Thermo-sensitive	Odontoblast	Heat sensing ion channels TRPV1? TRPV2? TRPV3 TRPV4 TRPM3	Heat-induced dental pain in healthy or pathological state (Odontoblast transducer theory)
		Cold sensing ion channels TRPA1 TRPM8	Cold-induced dental pain in healthy or pathological state (odontoblast transducer theory)
		TRAAK, TREK-1	K2p channels may play a role as thermos-sensors (Neural theory)
	DPA neurons	Heat sensing ion channels TRPV1 TRPV2 TRPV4 TRPM3 ANO1	Heat induced dental pain in healthy or pathol gical state (Neural theory)
		Cold sensing ion channels TRPA1 TRPM8	Cold induced dental pain in healthy or pathological state (Neural theory)
	Others PDL cells/Fibroblast	Thermosesing TRP channels	Function in dental pain sensing mechanism is not clear
Mechano-sensitive	Odontoblast	TRP channels TRPV1 TRPV2 TRPV4 TRPC1 TRPC6 TRPP1 TRPP2 TRPM7 TRPM3	Sensing movement of dentine tubular fluid (Hydrodynamic theory) or microdeformation of tooth structure
		Piezo channels PIEZO1? PIEZO2?	
		TREK1	
	DPA neurons	TRP channels TRPA1 TRPV1 TRPV2 TRPV4 TRPM3 TRPM7	
		Piezo channels PIEZO2	
		ASIC3	
Ligand-gated	Odontoblast	Purinergic receptors P2X4, P2X7	Paracrine or autocrine signaling molecule
	DPA neurons	Ligand gated ion channels P2X2, P2X3	Paracrine or autocrine signaling molecule
Voltage-gated	Odontoblast	Voltage gated ion channels Na_V_1.6 Ca_V_1.2 K_V_1.1 K_V_1.2	Role of voltage gated ion channels in odontoblasts is not clear
	DPA neurons	Voltage gated ion channels Na_V_1.6-9 Ca_V_1.2 K_V_1.1 K_V_1.2	Function in transmission of nociceptive information
